# Comparative analysis of machine learning approaches for predicting respiratory virus infection and symptom severity

**DOI:** 10.7717/peerj.15552

**Published:** 2023-06-30

**Authors:** Yunus Emre Işık, Zafer Aydın

**Affiliations:** 1 Department of Management Information Systems, Sivas Cumhuriyet University, Sivas, Turkey; 2 Department of Computer Engineering, Abdullah Gül University, Kayseri, Turkey

**Keywords:** Biology and genetics, Feature evaluation and selection, Machine learning, Pathway analysis, Respiratory infection prediction

## Abstract

Respiratory diseases are among the major health problems causing a burden on hospitals. Diagnosis of infection and rapid prediction of severity without time-consuming clinical tests could be beneficial in preventing the spread and progression of the disease, especially in countries where health systems remain incapable. Personalized medicine studies involving statistics and computer technologies could help to address this need. In addition to individual studies, competitions are also held such as Dialogue for Reverse Engineering Assessment and Methods (DREAM) challenge which is a community-driven organization with a mission to research biology, bioinformatics, and biomedicine. One of these competitions was the Respiratory Viral DREAM Challenge, which aimed to develop early predictive biomarkers for respiratory virus infections. These efforts are promising, however, the prediction performance of the computational methods developed for detecting respiratory diseases still has room for improvement. In this study, we focused on improving the performance of predicting the infection and symptom severity of individuals infected with various respiratory viruses using gene expression data collected before and after exposure. The publicly available gene expression dataset in the Gene Expression Omnibus, named GSE73072, containing samples exposed to four respiratory viruses (H1N1, H3N2, human rhinovirus (HRV), and respiratory syncytial virus (RSV)) was used as input data. Various preprocessing methods and machine learning algorithms were implemented and compared to achieve the best prediction performance. The experimental results showed that the proposed approaches obtained a prediction performance of 0.9746 area under the precision-recall curve (AUPRC) for infection (*i.e*., shedding) prediction (SC-1), 0.9182 AUPRC for symptom class prediction (SC-2), and 0.6733 Pearson correlation for symptom score prediction (SC-3) by outperforming the best leaderboard scores of Respiratory Viral DREAM Challenge (a 4.48% improvement for SC-1, a 13.68% improvement for SC-2, and a 13.98% improvement for SC-3). Additionally, over-representation analysis (ORA), which is a statistical method for objectively determining whether certain genes are more prevalent in pre-defined sets such as pathways, was applied using the most significant genes selected by feature selection methods. The results show that pathways associated with the ‘adaptive immune system’ and ‘immune disease’ are strongly linked to pre-infection and symptom development. These findings contribute to our knowledge about predicting respiratory infections and are expected to facilitate the development of future studies that concentrate on predicting not only infections but also the associated symptoms.

## Introduction

Respiratory infections are the leading cause of acute illnesses globally in both adults and children from past to present. According to a report by the World Health Organization (2020), respiratory-related infections cause nearly four million deaths per year. It is also one of the major diseases that threaten human health with high morbidity, severity, and medical costs (Yuan et al., 2022). The numbers are even higher especially in undeveloped and developing countries due to inadequate healthcare systems. Geographic differences and socioeconomic factors of the populations also affect the variation in viral etiology and the number of cases across countries (Liu et al., 2015).

Although numerous pathogens such as bacteria, fungi, mycoplasma, *etc*. can cause infection, a large proportion of respiratory infections is caused by viruses. HRV has been identified as the virus most commonly associated with respiratory diseases, accounting for about 40% of infections. Influenza viruses, RSV, and Coronavirus follow HRV in terms of frequency (Lambkin-Williams et al., 2018). These pathogens all have similar clinical symptoms but sometimes require completely different treatments. Otherwise, severe pneumonia may develop, which can cause mortality or some complications.

Most infections result in mild symptoms such as runny nose, sore throat, and headache, but some individuals remain asymptomatic despite exposure to respiratory viruses (Jansen et al., 2011; Byington et al., 2015). This was commonly reported by people during the period of COVID. Some COVID patients went through the disease with severe symptoms despite being in the best of health before infection, while some chronically ill elderly people showed no symptoms (Zhang et al., 2020; Esteban et al., 2021). The host response following the exposure is linked to genetic predisposition, disruption of the individual’s microbiome (Pichon, Lina & Josset, 2017), being in high-risk group (Walker et al., 2022) and effective immune surveillance. However, the variation in the physiological responses of people to viral exposure is poorly understood. The lack of understanding about the precise physiological or genetic factors delays the detection of infection, which leads to the spreading of the virus and thereby increases the death toll. On the other hand, many of the processes that lead to these variations occur in the peripheral blood through the activation and recruitment of circulating immune cells (Heidema et al., 2008). Hence an idea arises as to whether or not markers of susceptibility and resistance to infection may be identified in blood samples.

A lot of studies have focused on the idea of using both statistical and *in silico* methods to find out predictors of respiratory infection and make forecasting for individuals. Bongen et al. (2018) applied a meta-analysis to several data sets and observed that the expression of the KLRD1 gene in blood decreased after influenza virus infection. They were also able to predict the symptomatic and asymptomatic samples with an area under the receiver operating characteristic (AUROC) value of 0.91 in a validation set of H3N2 influenza samples. Barral-Arca et al. (2020) found 17 characteristic genes for RSV by applying logistic regression to 296 infected and 266 healthy samples from different datasets. ORA of these genes showed that immunological pathways such as the innate immune system and the adaptive immune system are closely associated with RSV. In a study by Xu et al. (2019), the OTOF and SOCS1 genes were identified as discriminators of HRV infections in machine learning experiments on gene expression profiles.

In a comprehensive study, different machine learning and feature selection methods were compared using three different datasets containing RSV-, HRV-, and influenza-infected samples (Radovic et al., 2017). The proposed model included a modified minimum Redundancy—Maximum Relevance (mRMR) method and a naïve Bayes classifier that achieved an average accuracy of 91% when the number of gene expression features is 40. The authors also applied an ORA on the top-50 genes selected by the best feature selection method and reported that all viruses are related to the immune response to viral infection.

Recently, deep learning-based models have also become popular in predicting respiratory virus infection. Zan et al. (2022) proposed a six-layer Deep Neural Network (DNN) model to predict whether a person would catch flu prior to exposure to Influenza A viruses. The model outperformed SVM, RF, and convolutional neural network, achieving a cross-validated AUPRC of 0.758 for DEE3 H1N1 and an AUPRC of 0.901 for DEE2 H3N2 experiments, respectively. In another study, a recurrent neural network achieved more than 90% prediction accuracy for predicting whether samples are infected with H3N2 (Tarakeswara Rao et al., 2022).

In addition to these efforts, a competition titled Respiratory Viral DREAM Challenge was held in 2016 by Sage Bionetworks, Duke University, and Defense Advanced Research Projects Agency (DARPA). DREAM is a community-driven organisation with the mission of advancing biomedical and systems biology research through crowdsourcing competition. Competitions usually focus on tackling a specific biomedical research question, narrowed down to a specific disease. As the competitions are open to researchers around the world, a wide range of ideas and solutions can be presented. This allows for the most effective solution to the problem being sought. The Respiratory Viral DREAM Challenge was one of these competitions which aimed to develop early predictors of susceptibility to and contagiousness of respiratory reactions based on gene expression profiles collected before and after exposure (Fourati et al., 2018). Participants were expected to make predictions for three different sub-challenges, including viral shedding, presence of symptoms, and severity, both before and after exposure. According to the results of the leaderboard stage, the proposed models achieved an AUPRC of 0.92 for predicting whether a person was infected, whereas obtained approximately an AUPRC of 0.78 for predicting the presence of symptoms. On the other hand, only a 0.53 Pearson correlation similarity score was obtained for continuous symptom severity prediction. Moreover, the heme metabolism pathway showed a strong relationship with the development of symptoms as a result of enrichment analyses of the susceptible genes identified by the participants.

In this study, we aimed to outperform the leaderboard scores of the DREAM challenge for all categories and phases by employing different pre-processing techniques and machine learning methods. Additionally, we have utilized a two-step feature selection method that leverages both wrapper and filtering algorithms to enable the identification of the most effective genes for both infection and symptom predictions. The implementation of a two-step approach allowed us to select the least number of genes (*i.e*., features) that yield the highest predictive performance. Thanks to this approach, we were able to identify common optimal gene subsets using ORA. This may provide a greater insight into the relationship between infection and symptom severity prediction. Furthermore, the pre- and post-exposure analyses also yielded valuable results that may be useful to other researchers for further studies on respiratory viruses. After conducting a literature review, we were unable to find any study that investigates the common sides of infection and symptom severity. Overall, our study is expected to provide significant benefits for future research in the field, especially for the development and improvement of predictive performance and statistical identification of biomarker genes.

## Materials and Methods

### Dataset

In our experiments, we used a public dataset on Gene Expression Omnibus (GEO) with accession number GSE73072, which was also used as the dataset for the leaderboard stage in the Respiratory Viral DREAM Challenge (Liu et al., 2016). GSE73072 includes datasets from seven related studies conducted by Duke University under contract with the DARPA Predicting Health and Disease program. Each of the experiments contains a different number of samples from one of four different respiratory viruses: H1N1, H3N2, HRV, or RSV. After aggregation, the experiments were referred to as RSV DEE1, H3N2 DEE2, H1N1 DEE3, H1N1 DEE4X, H3N2 DEE5, HRV UVA, and HRV DUKE, respectively. Abbreviations such as DEE1 or DEE2 indicate the names of the experiments and have not been changed in this article to avoid confusion.

To understand susceptibility to respiratory infections in humans, samples were collected both before and after infection. Therefore, peripheral blood samples were collected from healthy volunteers starting the day before (*i.e*., T.-24 or T.-30 h). Each volunteer was inoculated at time T.0 in a controlled environment with only one of the four different live respiratory viruses. Sampling began 1 day (24 or 30 h) before inoculation and continued at various intervals up to 7 days later. However, in this study, we only took into account up to 1 day after inoculation because one of the objectives of our study was to determine the early predictors immediately after exposure, which is the same as the objective of the DREAM challenge. To extract gene expression profiles from blood samples, an Affymetrix Human Genome U133A 2.0 microarray with 22,277 probes was used.

**Figure 1:**
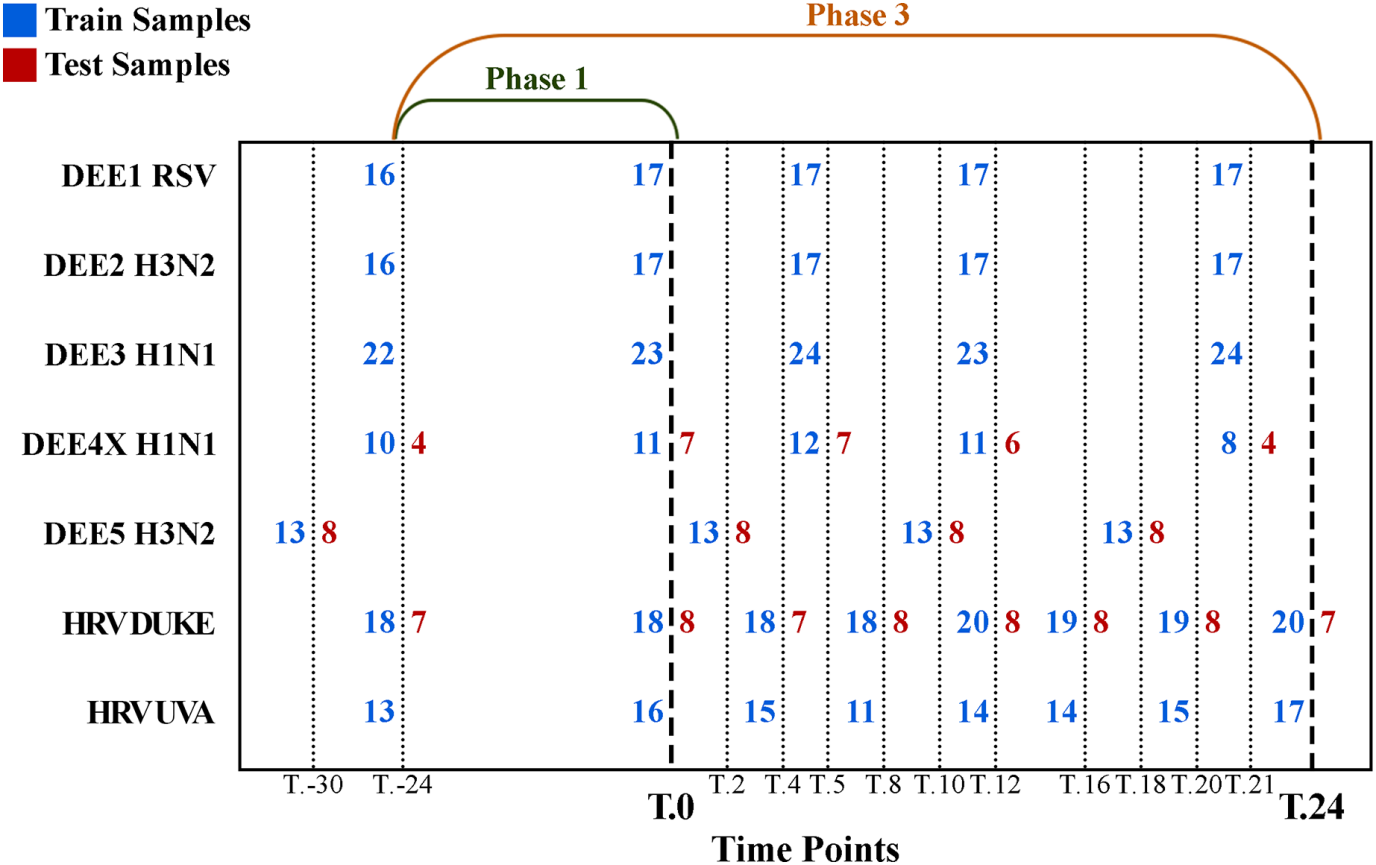
Detailed numbers of samples collected at different time points for each experiment. The y-axis shows abbreviations of the seven experiments (*i.e*., sub-datasets) included in the GSE73072 dataset. The blue and red numbers represent the number of training and test samples in related experiments, respectively. Phase 1 (up to T.0) and phase 3 (up to T.24) represent the prediction phases corresponding to the data prior to viral exposure and up to 24 h after exposure.

The number of samples collected at different time points is shown in Fig. 1, where rows denote experiments, columns denote time points, blue numbers denote training samples, and red numbers denote test samples. For example, while 21 samples were collected for the DEE5 H3N2 experiment prior to exposure at time T.-30 h, only 14 samples were collected for DEE4X H1N1 at time T.-24. The training and testing samples were chosen from the GSE73072 dataset by the community of DREAM Challenge. Therefore, we used the same test samples in our analyses to make a fair comparison between ours and the challenge results. Consequently, our dataset contains a fixed number of seven, eight, and eight test subjects to be predicted for DEE4X H1N1, DEE5 H3N2, HRV DUKE, respectively. Nevertheless, it should be noted that predictions will be computed for these subjects both before and after exposure.

To determine whether a subject is infected, lavage particles from the nasal passages were analyzed in clinical settings. If the particles showed evidence of viral infection, shedding of the sample was labeled as 1; otherwise, it was labeled as 0. In addition, subjects were asked to rate the severity of eight different symptoms, including runny nose, headache, malaise, myalgia, sneezing, sore throat, and nasal congestion, at regular intervals on a 0–4 scale. These scores were used to calculate the Jackson score (Jackson et al., 1960), which is known as the best method for measuring symptom severity. If the score was less than 6, the presence of the symptom was labeled as 0, indicating that the sample was asymptomatic; otherwise, the sample was labeled as 1. The log10 transformation of the Jackson score has also been used to represent continuous symptom severity.

### Motivation and problem definition

Respiratory viral infections are still one of the most common diseases imposing an economic burden on hospitals. Diagnosing as early as possible reduces mortality rates and contributes economically. At this stage, artificial intelligence-based systems are one of the solutions. However, since the viruses that cause respiratory diseases are spread through airborne transmission, it is difficult to determine the exact time of infection and onset of the symptoms from a genetic perspective. This makes it difficult to identify early markers of infection. To address these needs Respiratory Viral DREAM Challenge was held in 2016, which stands out from many studies in the literature in terms of the use of data sets containing various types of viruses, evaluating symptom severity, and including exact time when the subject is exposed to virus. Participants of the challenge were expected to make predictions for two phases, pre-exposure (phase 1) and post-exposure (phase 3), on data sets generated by injecting different respiratory viruses into volunteer subjects. Participants were expected to make predictions in three different tasks:

- *Sub-Challenge 1 (SC-1)*: Prediction of viral shedding, *i.e*., whether the individual is infected or not. A binary outcome to evaluate infection prediction rate. Aims to find out predictors that cause infection.
- *Sub-Challenge 2 (SC-2)*: Prediction of symptomatic response to exposure. In other words, predicting whether or not the subject will become symptomatic after exposure. Aims to find out predictors that cause severe symptoms.
- *Sub-Challenge 3 (SC-3)*: Continuous-valued prediction of symptom score. Since the discrete-valued symptom score is calculated using the Jackson score, this task includes the direct prediction of the log-transformed version of the Jackson score. Aims to find out predictors that cause severe symptoms.

To form the datasets, gene expression profiles of each sample had been obtained using a microarray with 22,277 probes, each representing one or more genes. These gene expression values were obtained by collecting blood samples at different time points and constitute the input features for the prediction models.

For each of the three sub-challenges, participants had first made their submissions for the test set of the leaderboard phase. Then, in the second phase, an independent test set was used to evaluate the performance of the submissions. In addition to developing models with high prediction performance, the goal of this challenge was to identify predictors of infection as well as symptoms for both pre- and post-exposure periods. Table 1 shows the best performing submissions for the leaderboard phase of the challenge. The results show that there is still room for improvement especially for SC-2 and SC-3.

**Table 1:** Best performing leaderboard scores of the Respiratory Viral DREAM Challenge. SC represents different sub-challenges.

Time index	AUPRC	AUROC	Pearson correlation
**SC-1**			
T.24 (phase 3)	0.9298	0.8137	–
T.0 (phase 1)	0.9247	0.8039	–
**SC-2**			
T.0 (phase 1)	0.7814	0.7348	–
T.24 (phase 3)	0.7511	0.7348	–
**SC-3**			
T.0 (phase 1)	–	–	0.5335
T.24 (phase 3)	–	–	0.5

The main motivation of this study is to improve the prediction performance of the challenge results using pre-processing and machine learning methods. There are multiple reasons for focusing on the results of the DREAM challenge in this study. First of all the DREAM challenge included multiple prediction tasks with varying objectives, all of which utilized the same gene expression data. Based on that the challenge dataset allowed us to perform a comprehensive analysis on different prediction tasks. Second, there is no other publicly available dataset published after DREAM challenge that contains the four different respiratory viruses along with actual class label information. Third, sampling of gene expression had started before the exposure, which led to the opportunity to perform pre-infection analysis. This way, we were able to propose models specifically for pre- and post-exposure data as well as various prediction problems. Fourth, the possibility of identifying related common genes that have a role in both infection and symptom development was another motivation for this study. Finally, the results obtained in the DREAM challenge are still open for further improvement, which shows that the prediction problems are not solved yet. It should be noted that there are limited studies in the literature that perform a comprehensive analysis similar to this work using data for multiple viruses and experiments, data for multiple time-points, computing predictions for pre-exposure and post-exposure periods, and finding predictors (*i.e*., genes) that are important for infection and symptom development.

### Prediction algorithms

As explained in the problem definition, SC-1 and SC-2 are binary classification problems. Classification algorithms can use gene expression values as input to make a prediction about whether a subject is infected or not. In our experiments, Logistic Regression (LR), Support Vector Machine (SVM), Random Forest (RF), k Nearest Neighbors (kNN) (Crisci, Ghattas & Perera, 2012) and XGBoost (XGB) (Chen & Guestrin, 2016) were employed as classification methods, where RF and XGB are ensemble methods that combine multiple learners to obtain a combined model that can outperform its base learners.

On the other hand, the goal of SC-3 is to estimate the severity of symptoms for a given subject, which is represented as a continuous-valued score. Therefore, SC-3 is a regression problem. The Lasso, Elastic Net, Ridge (Steinauer et al., 2021), Linear Support Vector Regression (SVR), Gradient Boosting Regressor (Madhuri, Anuradha & Pujitha, 2019), K Neighbors Regressor (KNN R.), Decision Tree Regressor (El Sayed et al., 2022), XGBRegressor (Tahseen & Danti, 2022) and Bayesian Ridge (Shi, Abdel-Aty & Lee, 2016) methods were used for the SC-3 problem.

### Hyper-parameter optimization

One of the factors that lead to high predictive performance for machine learning methods is the proper tuning of hyper-parameters. When the hyper-parameters of an algorithm are tuned properly, the prediction accuracy can be increased. In our study, each model training and testing experiment was conducted with both optimized and non-optimized models depending on whether hyper-parameters are optimized or not. In the experiments, we used the open-source library named Optuna (Akiba et al., 2019) to optimize the hyper-parameters, which performs a random search and finds an optimal subset of hyper-parameter values by evaluating the assigned parameters.

Since the number of samples is small even for training data, the leave-one-out-cross-validation (LOOCV) technique was preferred during parameter optimization. In each iteration, one sample is marked as validation data and the rest is used to train the model with the specified parameter set. In the end, a prediction is obtained for each sample and the final accuracy is computed by averaging predictions obtained for all samples. This accuracy indicates the performance of the parameter set. The LOOCV is repeated for all hyper-parameter combinations sampled using random search and the particular hyper-parameter set that gives the best LOOCV performance is selected as the optimum. To find the best values of hyper-parameters, the overall accuracy is optimized for SC-1 and SC-2 and the Pearson correlation for SC-3. The hyper-parameter optimization steps explained above were performed for all pre-processing methods, *e.g*., feature selection, virus merging, and the results for optimized and non-optimized versions of the models were reported. Details of parameter spaces of the algorithms are given as a supplementary file (Supplemental 1).

### Data preprocessing

Our main goal is to predict the subject’s infection, symptom presence, and symptom severity as accurately as possible. In this article, we proposed machine learning-based models that take gene expression profiles of subjects as input and make forecasting about infection and symptoms. All methods, pre- and post-processing codes were implemented using the Python programming language. To implement classification and regression algorithms, we used the open-source machine learning library of Python called scikit-learn (Pedregosa et al., 2011). To implement feature selection methods, we used the scikit-feature library of Python (Li et al., 2017).

The sampling process was not performed in all time spans for each subject, which causes missing value problem. For example, blood samples of subjects with IDs “3013” and “3015” were not collected at the T.-24 time point, nor the samples of subject “3014” on T.0. Consequently, for a given experiment, the number of samples at different time points may not be equal, and such unbalanced sample numbers must be considered in experimental analysis so that machine learning models can be trained and tested systematically in a way that combines information from multiple time-points. To address this issue, those time points, which do not include data for all subjects of a given phase and experiment or those subjects who do not have data in all time points of a given phase and experiment could have been excluded from the analysis. However, to allow a fair comparison between the challenge results and our proposed models, neither subjects nor time points were discarded. Instead, we propose single time point and experiment (STPE), and average of features (AF) approaches to process data from all subjects and time points.

Our experiments include two main stages. In the first one, machine learning models are applied only to preprocessed datasets obtained using STPE, AF, and/or virus merge (VM) approaches, which are explained below in more detail. This stage shows the prediction performance of the full use of gene expression profiles. The second stage consists of applying feature selection for the prediction of respiratory infection and the determination of significant genes that have an impact on the prediction of infection and symptoms.

#### Single time point and experiment (STPE) approach

Samples related to each time point of the experiment are treated as a separate dataset in the STPE approach. Machine learning models are trained separately for each dataset belonging to a particular time point and experiment. After training, for each experiment in the test set, the class probability distributions of the subjects are predicted for each time point in each phase (*i.e*., phase 1 or phase 3) because our goal is to make a prediction for a phase that spans multiple time points, rather than for a specific time point. The final class distributions were obtained by averaging these probabilities obtained for different time points. If gene expression data are not available for a subject at particular time points, these time points are excluded and the distributions obtained for the remaining time points are used to calculate the average. For SC-3, instead of a class probability distribution, symptom severity is predicted and averaged to calculate the phase prediction for each subject. As described above, time points when the subject has no gene expression samples are ignored. The STPE approach allows us to use data for all subjects and time points available for a given phase and experiment.

**Figure 2:**
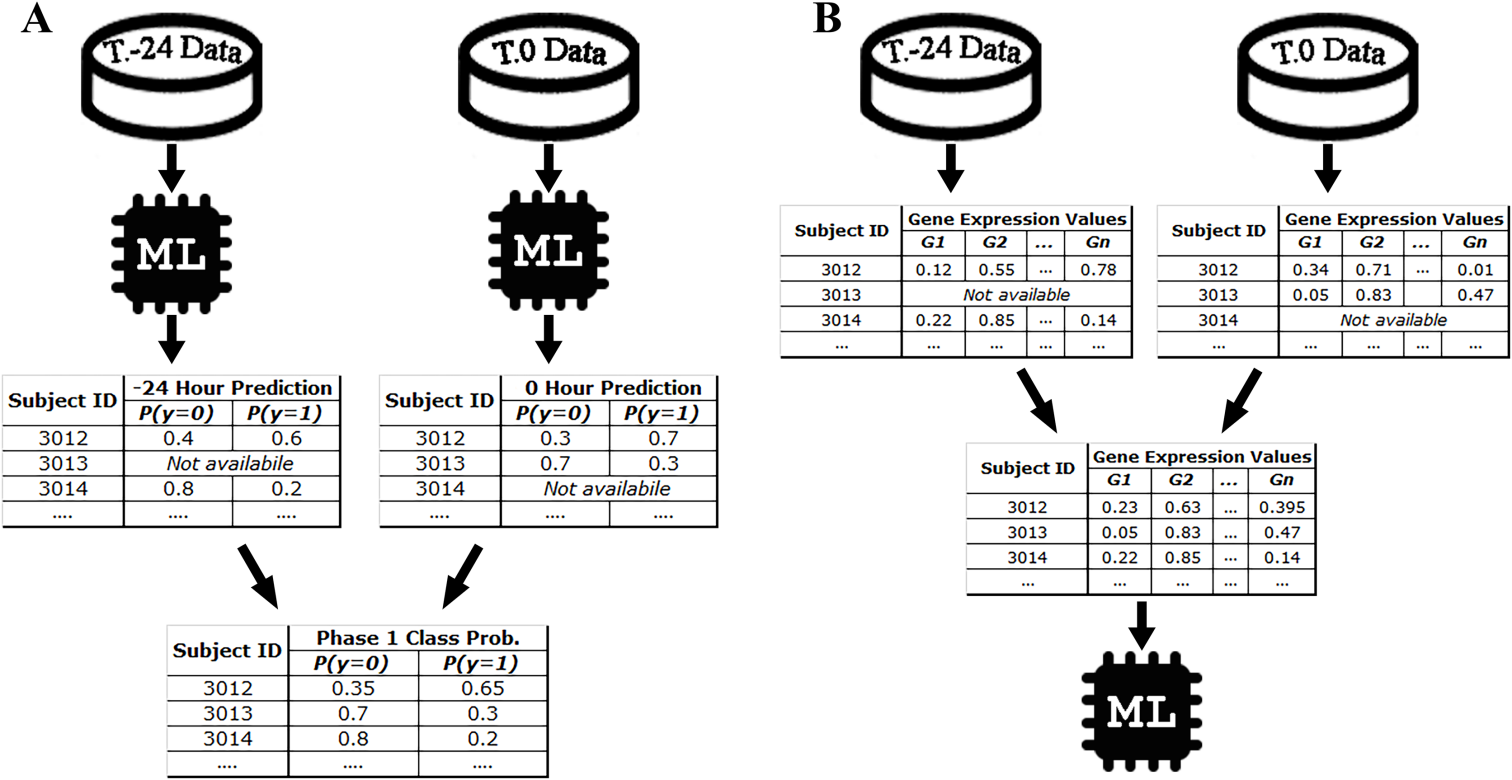
Class probability calculation in STPE (A) and AF (B) Approaches. STPE approach takes the average of class probabilities from different time points predicted by machine learning algorithms. AF takes the average of gene expression data from different time points. Both approaches use all samples and time points available without discarding any of them.

For example, since there are 10 samples of DEE4X H1N1 in training set at time T.-24, a separate model is trained for SC-1 using these samples only. Then the class probabilities of the four test set subjects belonging to DEE4X H1N1 are predicted using the gene expression data of these subjects as input. The same process is repeated for time point T.0 with 11 training samples and seven test samples, because a different number of samples are collected in T.0. Since only time points T.-24 and T.0 are included in phase 1 for DEE4X H1N1, probabilities from two different time points are averaged to calculate the final class probabilities of phase 1 (see Fig. 2).

#### Average of features (AF) approach

In the AF approach, the average of the input feature vectors at different time points for each subject is used as input to the ML models. If a subject does not have gene expression data at a particular time point, that time point is ignored and only the average of the available feature vectors is computed. Figure 2 shows an example of the steps in the AF approach. For example, the subject with ID 3013 has gene expression data at time point T.0 but does not have data at time point T.-24. Therefore, there is only one time point for this subject to make a prediction for phase 1. On the other hand, the subject with ID 3012 has gene expression data both for time points T.0 and T.24. Rather than just one time point, use of all available time points would be more effective, as more information makes machine learning models more robust in computing predictions.

The AF approach simply uses the average of the gene expression profiles and hence time point information is ignored. Although this may be considered as a downside of this approach, from another perspective it may also be an advantage, since the timing of symptoms varies from person to person and even virus to virus. For example, phase 3 contains eight different time points in the HRV DUKE experiment. While some subjects may become symptomatic between time points T.4 and T.12, others may show symptoms after T.12. As machine learning models cannot be trained/tested for each subject individually depending on the time point, symptom signals from all subjects should be acquired in a generalized model. This is because we assume that the changes in gene expression also begin with the onset of symptoms, making it easier to capture the changed signals (*i.e*., gene expression) by machine learning. In this way, despite the fact that gene expression signals of the subjects can be weak or strong at different time points, distinctive signals can be captured for all subjects using the AF approach, which also facilitates the identification of key gene expressions that impact disease prediction.

#### Virus merge (VM) approach

Since machine learning and pattern recognition derive their strength from data, problems of under- or over-fitting can arise when applying machine learning to small data sets (Vabalas et al., 2019). The larger the number of samples in training set, the more robust the models become in predicting new samples. However, the sample size of our training dataset is quite small compared to the typical size of the datasets used to train machine learning models. Therefore, in the VM approach, different experiments containing the same virus family were merged to increase the size of the training datasets. For this purpose, the training sets for the following experiments were merged: DEE4X H1N1 and DEE3 H1N1, DEE5 H3N2 and DEE2 H3N2, HRV UVA, and HRV DUKE. For example, in order to compute predictions for the four test samples of DEE4X H1N1 at T.-24, the model will be trained with 32 samples corresponding to 22 samples of DEE3 H1N1 and 10 samples of DEE4X H1N1 at the time point of -24 in the VM approach. Since VM is a preprocessing approach, it can also be used in combination with the other proposed approaches AF and STPE. This allows us to increase the size of training sets used to train machine learning models for all the approaches proposed.

#### Feature selection

Feature selection (FS) is defined as the process of eliminating redundant and irrelevant features from a dataset to improve the performance of a learning algorithm (Liu & Motoda, 1998). Thus, not only does FS help reduce the number of dimensions, but it can also improve the predictive performance. Machine learning-based FS methods can be divided into three main categories: filtering, wrapper, and embedding methods (Işık et al., 2021). Filtering approaches assign a score to each feature and rank them to find the optimal feature set by scoring each or a subset of features based on various measures such as mutual information, similarity, or correlation. Then, these ranked features whose scores are below the threshold are eliminated. Wrapper methods, on the other hand, include a learning algorithm for evaluating feature subsets. The optimal subset is selected depending on the performance of the prediction model. Therefore, wrapper-based feature selection methods are also called classifier-dependent approaches.

Our proposed two-step FS method includes both the filtering and wrapper approaches. The process starts with applying a filtering method to the training set. During the filtering step, the correlation value of each feature (*i.e*., gene expression) is calculated. Then, the features are sorted in descending order by their correlation score, and the training set is re-arranged according to the new order of the features. The second step aims to find the best subset of features. For this reason, starting from the most highly correlated feature, a subset is formed by adding the next feature at each iteration. The performance of each subset is evaluated using a wrapper algorithm and a LOOCV experiment on training set. Similar to hyper-parameter optimization, for SC-1 and SC-2, the overall accuracy and for SC-3 the Pearson correlation coefficient are optimized as the performance metrics to find the best feature subset. Since the main objective of feature selection is to reduce the number of dimensions, the least number of features that achieved the highest predictive performance was marked as the optimal subset of features. For example, if the first three and the first 20 features achieve the same highest accuracy as 75%, the first three features are selected as the optimal set. Once the best feature subset is found using training set, the test set is re-ordered using these features.

**Figure 3:**
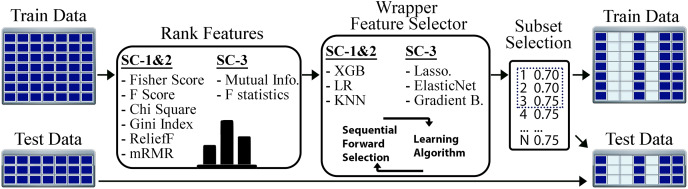
Steps of feature selection process. Features in training data were ranked by filtering metrics and evaluated by a wrapper approach. All steps are applied only to training data to evaluate and select features. After finding the best feature subset training and testing samples are re-arranged using the features selected.

The flow of our feature selection approach is given in Fig. 3. For the STPE approach, FS is performed separately for each experiment and time point. For the AF approach, FS is performed for each experiment separately after the feature vectors are averaged for the given phase. After rearranging the training and test sets using the selected subset of features, model training and testing experiments were performed for STPE, AF and VM approaches.

To find the best algorithms for both filtering and wrapper approaches, we compared different methods. Fisher score, F-score, Chi-square, RelieF, mRMR were used as filtering approaches for the problems SC-1 and SC-2. These methods calculate distinctiveness scores of a feature for a categorical class label. On the other hand, for SC-3 which is a regression problem, the mutual information (MI) and F-statistics were used to calculate the degree of correlation between a given feature and the output label. Similar to filtering step, wrapper algorithms also differ based on the sub-challenges. While XGBoost, LR, and KNN were employed for SC-1 and SC-2, Lasso, ElasticNet and Gradient Boosting Regressor were used for SC-3 as the prediction algorithms for the wrapper method.

## Results

As explained in the introduction and problem definition sections, the main goal of this study is to propose machine learning models that achieve better prediction performance than the best performing methods of Respiratory Viral Dream Challenge (Fourati et al., 2018). Detailed leaderboard scores obtained by the participants of the challenge are shown in 1. Our results are divided into three different tables to show the performance for classification and regression problems.

Table 2 contains the results for data pre-processing approaches (*i.e*., STP, AF, and VM). This table only includes our results that are better than the best-performing leaderboard results of the challenge. It can be concluded that the data pre-processing approaches were able to produce better prediction scores than the leaderboard results in all subchallenges and phases. In particular, AF-based models, obtained the highest values, especially in post-exposure prediction (*i.e*., phase 3). Moreover, although the highest AUPRC was 0.75 for the SC-2 phase 3 category of the challenge leaderboard, our AF models achieved a much higher score with an AUPRC of 0.92.

**Table 2:** Results of data pre-processing methods that outperformed the leaderboard scores for all sub-challenges.

Pre process	Classifier	AUPRC	AUROC	Pre process	Regressor	Pearson	MSE
**SC1-Ph.1**				**SC3-Ph.1**			
STPE VM*	LR	0.9328	0.7843	STPE	LinearSVR	0.5897	0.2273
*LB*.	–	*0.9247*	*0.8039*	STPE VM*	KNN R.	0.5608	0.1969
**SC1-Ph.3**				STPE VM	KNN R.	0.5593	0.1987
AF*	RF	0.9541	0.8676	STPE*	Bayes R.	0.5300	0.2000
STPE VM*	LR	0.9506	0.8627	STPE*	Ridge	0.5299	0.2001
AF VM*	XGB	0.9395	0.8431	STPE	Ridge	0.5296	0.2001
STPE VM*	RF	0.937	0.848	AF	Ridge	0.5274	0.1983
*LB*.	*-*	*0.9298*	*0.8137*	*LB*.	*-*	*0.5335*	–
**SC2-Ph.1**				**SC3-Ph.3**			
AF*	LR	0.8522	0.8485	AF VM*	KNN R.	0.5963	0.1889
STPE*	LR	0.7886	0.7652	AF*	KNN R.	0.5948	0.1891
*LB*.	–	*0.7814*	*0.7348*	STPE VM*	Gradient R.	0.5947	0.1965
**SC2-Ph.3**				STPE VM*	D.Tree R.	0.5906	0.1901
AF*	LR	0.9182	0.9015	AF VM	KNN R.	0.5824	0.1822
AF*	KNN	0.8517	0.8182	STPE VM*	XGB R.	0.5646	0.2158
AF VM*	KNN	0.8517	0.8182	STPE*	KNN R.	0.5449	0.2000
STPE*	LR	0.8101	0.803	STPE	XGB R.	0.5343	0.2265
STPE VM*	KNN	0.8045	0.7803	STPE VM*	KNN R.	0.5195	0.2070
*LB*.	–	*0.7511*	*0.7348*	*LB*.	–	*0.5*	–

**Note:**

Left side contains SC-1 and SC-2 (classification problems), and right side contains performance results of SC-3 (regression problem). An asterisk (*) indicates that the hyper-parameters were not optimized. Italic values (LB) show the best results for sub-challenges and phases of the Respiratory Viral DREAM Challenge.

Because SC-3 was associated with continuous symptom severity, the predictive performance of the models was evaluated using Pearson correlation coefficient, which describes the strength of the linear relationship between two variables. While a correlation of one indicates an exact linear relationship, 0 represents no relationship or similarity between the variables. In addition, we also calculated the mean square error (MSE) for each model. This is because the MSE is one of the most well-known methods of measurement, especially in regression problems, and could also be informative.

Among our proposed models for SC-3, LinearSVR with the STPE approach achieved a Pearson correlation of 0.5897 for pre-exposure prediction. For SC-3 phase 3, our models also achieved higher values than the best-performing models of the DREAM challenge (a Pearson correlation of 0.5963). When the results of SC-3 are evaluated by MSE, the kNN regressor based on AF VM scored the lowest with an MSE of 0.1822. Even though MSE is expected to take low values when Pearson coefficient takes high values, this is not always observed in our results. This is because while the Pearson coefficient measures the strength of the relationship between the two variables, the MSE expresses the overall error of the model.

If we compare data pre-processing methods (*i.e*., STP, AF, and VM) and prediction methods (*i.e*., LR, RF, SVM, k-NN, XGBoost, *etc*.), there is no winner that performs the best in all prediction tasks and phases. Furthermore, hyper-parameter optimization did not always improve the prediction performance of the models. This could be because the number of samples in the training set is small. Consequently, our proposed approaches achieved improvements of 1–3%, 7–16%, and 5–9% for SC-1, SC-2, and SC-3, respectively.

FS is often used not only to reduce the number of dimensions but also to find the features that are most important for classification. Therefore, selected gene expression probes can also be interpreted as significant genes that are important for infection and symptom severity. Table 3 shows the performance results of feature selection methods for SC-1 and SC-2. The “Wrapper” column indicates the classifier used by the forward selection algorithm, while the “Classifier” column indicates the classifier that is trained and tested for the prediction task after performing FS.

**Table 3:** Results of FS Methods for SC-1 and SC-2.

Feature selection	Pre process	Classifier	Wrapper	Number of features	AUPRC	AUROC
**SC-1 Ph.1**						
ReliefF*	STPE	LR	KNN	55	0.9341	0.8235
ReliefF	AF	LR	XGB	10	0.9264	0.7745
**SC-1 Ph.3**						
Fisher Score*	STPE	KNN	XGB	60	0.9746	0.9167
F Score*	STPE	KNN	XGB	60	0.9746	0.9167
mRMR*	STPE	SVM	XGB	275	0.9706	0.9118
mRMR*	STPE	KNN	KNN	6,805	0.9632	0.8775
Fisher Score*	STPE	RF	LR	20,481	0.9628	0.8725
Gini Index*	STPE	KNN	KNN	12,302	0.9572	0.8627
ReliefF*	STPE	XGB	KNN	18,913	0.9502	0.8725
ReliefF	STPE	LR	LR	22,277	0.9498	0.8627
Fisher Score*	AF	KNN	XGB	40	0.9429	0.8235
mRMR	AF	SVM	KNN	16	0.9325	0.7745
**SC-2 Ph.1**						
Fisher Score*	AF	KNN	KNN	17,566	0.8515	0.8712
F Score*	AF	KNN	KNN	17,566	0.8515	0.8712
Gini Index*	AF	LR	KNN	14,673	0.8365	0.8561
Chi Square*	AF	XGB	LR	8	0.8187	0.7765
Chi Square	STPE	KNN	XGB	54	0.8112	0.7689
Chi Square	AF	LR	KNN	5	0.8039	0.7879
**SC-2 Ph.3**						
Fisher Score*	AF	KNN	KNN	18,084	0.8956	0.8561
F Score*	AF	KNN	KNN	18,084	0.8956	0.8561
Gini Index*	AF	SVM	LR	116	0.8908	0.8939
Chi Square	AF	LR	LR	19	0.8759	0.8712
Chi Square	AF	LR	KNN	6	0.8675	0,8561
Chi Square	STPE	KNN	XGB	180	0.8595	0.8333
ReliefF	STPE	KNN	LR	12,206	0.8518	0.8447
Fisher Score*	STPE	KNN	KNN	22,214	0.8497	0.8598
Gini Index*	AF	LR	KNN	8,495	0.8462	0.8333
Gini Index*	AF	SVM	XGB	4	0.8428	0.8258
ReliefF	AF	LR	XGB	92	0.821	0.8106

**Note:**

After the features are ranked by a filtering approach, a wrapper algorithm is utilized to select the best feature subset. *Wrapper* column indicates the prediction algorithm used in wrapper method. *Number of Features* column represents the number of distinct features selected. An asterisk (*) indicates that the hyper-parameters were not optimized.

The “Number of Features” column represents the total number of gene expression features selected and used in the FS-based models. For the STPE approach, this value is calculated by summing the features selected at each time point in the given phase. For the AF approach, it is equal to the number of features selected after taking the average of the feature vectors from multiple time points. For example, for the STPE approach, the number of selected genes is 1 for DEE4X H1N1, 5 for human rhinovirus Duke (HRV Duke), and 1 for DEE5 H3N2 at time T.-24, whereas this number is 1 for DEE4X H1N1 and 47 for HRV Duke at time T.0 for the ReliefF models. Because phase 1 includes time indices prior to and including T.0, the total number of unique features used in the ReliefF experiment of SC-1 phase 1 became 55 after removing duplicates (some features may be selected repeatedly in multiple time points and/or experiments).

The model that used the AF-approach for data pre-processing, chi-square method for FS and LR as the classifier achieved an AUPRC of 0.8187 in the SC-2 phase 1 category, although only 8 gene expression features were used. Considering that the total number of features in each time point is 22,277, it can be interpreted that this model achieved a reasonably high score despite the small number of features. In addition, the Fisher score-based models achieved the best performance among all models using only 60 gene expression features in SC-1 phase 3 category. Similarly, it can be concluded that FS approaches highly improved the prediction of symptom severity scores with a Pearson correlation of 0.67, as shown in Table 4. When the performances of the models that employed FS are compared to the performances obtained without FS, reducing the number of features further improved the prediction performance except for SC-2 phase 3. FS-based models achieved between 1% and 17% improvement in AUPRC, depending on subcategory and phase.

**Table 4:** Results of FS methods for SC-3.

Feature selection	Pre process	Regressor	Wrapper	Number of features	Pearson	MSE
**SC-3 Ph.1**						
F Statis.	AF	Decision Tree R.	Gradient B.	259	0.5990	0.2357
Mutual Info.*	STPE	KNeighbors R.	ElasticNet	121	0.5566	0.1893
Mutual Info.*	STPE	KNeighbors R.	Gradient B.	979	0.5443	0.1917
F Statis.	STPE	Decision Tree R.	Lasso	404	0.5346	0.1998
**SC-3 Ph.3**						
F Statis.*	STPE	LinearSVR	ElasticNet	1,736	0.6733	0.1920
F Statis.	STPE	ElasticNet	ElasticNet	1,736	0.6073	0.1973
F Statis.	STPE	LinearSVR	ElasticNet	1,736	0.6069	0.2062
F Statis.	STPE	XGB Regressor	Lasso	1,526	0.5693	0.2114
Mutual Info.*	STPE	LinearSVR	Lasso	4,410	0.5576	0.2021
F Statis*	STPE	Ridge	ElasticNet	1,736	0.5507	0.2148
F Statis.*	AF	KNeighbors R.	ElasticNet	671	0.5455	0.1990

**Note:**

*Number of Features* column represents number of distinct features selected. An asterisk (*) indicates that the hyper-parameters were not optimized.

Evaluating the significance level of research results often involves utilizing statistical tests. Usually, these tests are considered reliable and appropriate when the sample size is more than 30 (Chang, Huang & Wu, 2006). Despite the relatively small sample size of 23 in our study, we performed a Z-test with a confidence level of 0.90 for the best performing models listed in the tables. Calculated Z-Scores and *p*-values are shared as supplementary file (Supplemental 2). Based on these results most of the improvements obtained in this article are not found to be statistically significant but it should be noted that these tests are performed with insufficient number of samples and therefore the test results could be unreliable.

**Figure 4:**
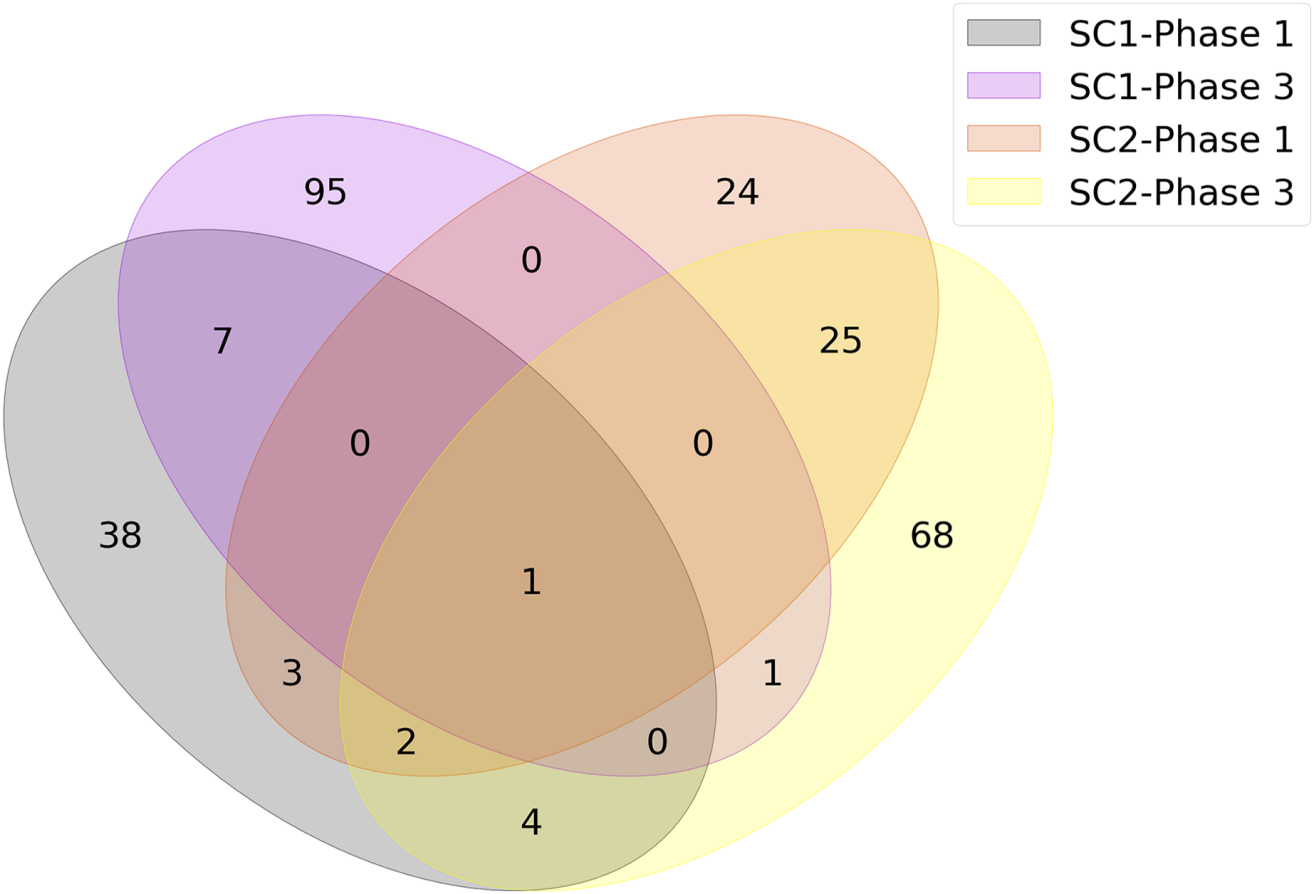
Number of genes jointly selected by the feature selection algorithms for the presence of viral shedding (SC-1) and the presence of symptoms (SC-2). The intersections of the clusters represent the selected genes for mutual genes for different challenges and phases. There is only one gene, *ATP7A*, as shown in the middle of the diagram, which has a strong discriminatory effect in terms of predictive performance for all sub-challenges and phases.

The union of genes selected by FS methods are shown in Fig. 4 as a Venn diagram according to different sub-challenges and phases. Because one of the main objectives of our analysis is to determine the genes that are important for infection, only models that selected less than 100 features were considered in constructing the diagram. Since each method could select different genes and any of them could be significant, the genes selected by different FS methods were combined as a single list for each sub-challenge and phase. This allowed us to identify the gene subset that achieved the highest predictive performance with the smallest number of features. As a result, the number of genes at the intersection of SC-1 phase 1 and SC-2 phase 1 (*i.e*., those that are common to SC-1 and SC-2 in phase 1) is 6, and this number is 2 for the genes that are common to SC-1 and SC-2 in phase 3, respectively. When evaluated according to sub-challenge, 8 genes were selected as common to SC-1 phase 1 and SC-1 phase 3 (*i.e*., those that are selected in SC-1 both for phase 1 and phase 3); 28 genes were at the intersection of SC-2 phase 1 and SC-2 phase 3. In addition, only 1 gene was selected for all sub-challenges and phases, namely “ATP7A”.

Despite the paucity of overlap among common genes, ORA was performed on the union of intersecting genes to gain better insight into the underlying association of genes with specific biological pathways. ORA is a simple statistical approach that determines which biological functions or processes (or pathways) are significantly enriched among genes in a given list (Tsuyuzaki et al., 2015). The degree of enrichment is expressed as a *p*-value calculated using a hyper-geometric test (or Fisher’s exact test) indicating whether terms are found in the gene lists more frequently than expected by chance. A *p*-value less than 0.05 is typically considered to be statistically significant.

To perform ORA, we used the WebGestalt platform, a web-based toolkit that takes a gene list as input and performs a functional enrichment analysis to make an interpretation of the given list (Liao et al., 2019). Because the pathways in different databases can differ in many ways, such as the number of pathways present, the size of the pathways, and how the pathways are curated, we used two well-known databases in our analysis: KEGG, and Reactome (Ogata et al., 1999; Gillespie et al., 2022).

Because we want to extract the underlying biological factors before and after exposure and the reasons for the symptoms, we need to analyze each sub-challenge and phase separately. Therefore, the intersecting genes for SC-1, SC-2, phase 1, and phase 3, whose numbers are listed in Fig. 4, were determined and used separately in ORA. For example, to analyze only SC-1, the intersecting genes of SC-1 phase 1 (orange circle in Fig. 4) and SC-1 phase 3 (pink circle in Fig. 4) were used as input.

**Figure 5:**
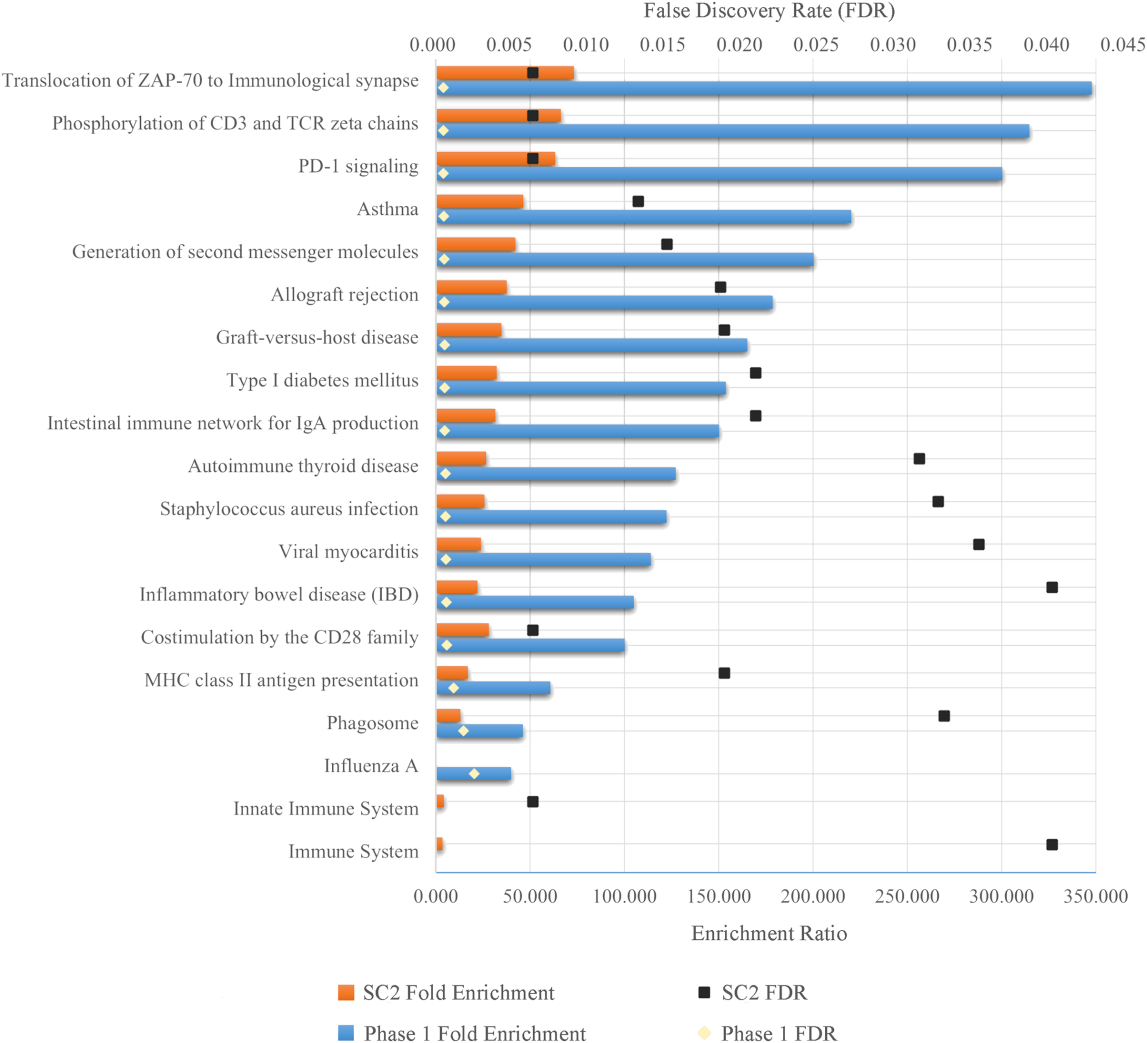
Enriched pathways as a result of over-representation analysis using intersecting genes from SC-2 and phase 1. The bars show the enrichment values of each listed pathway, whereas the black and gold dots indicate the false discovery rate of these values. The higher the FDR, the lower the confidence in the pathway. Since there is no enriched pathway in the other sub-challenges and in phase 3, they are not listed in the figure. Majority of enriched pathways were related to either *Adaptive Immune System* or *Immune Disease*.

As a result of ORA, the ratio and false discovery rate (FDR) of the enriched pathways for SC-2 and phase 1 are shown separately in Fig. 5. However, because the FDR values of the enriched pathways for SC-1 and phase 3 were above 0.05, the result obtained would not be significant and would be unreliable, and therefore only the pathways with FDR <0.05 were considered.

As can be seen in the Fig. 5, despite the fact that each FS method selected different number of genes in different sub-challenges and phases, mostly similar pathways are enriched. In particular, translocation of ZAP-70 to the immunological synapse, phosphorylation of CD3 and TCR zeta chains, and PD-1 signaling pathways are the most enriched pathways with the lowest FDR values. The majority of these pathways is that they are all part of the Adaptive Immune System group in the Reactome database. In addition, all other enriched pathways also have an association with either the immune system or immune diseases, with the exception of *Type I Diabetes Mellitus*, *Intestinal Immune Network for IgA Production*, *Viral Myocarditis* and *Phagosome*. ORA found out that HLA-DQA1, HLA-DQA2, HLA-DRB4 for phase 1 and HLA-DQB1, HLA-DRB4, HLA-DQA1 for SC-2 were the genes that had the maximum overlap with the enriched pathways.

In addition to these analysis, genes that are commonly selected among different experiments are also explored. Although the viruses in our experiments are different, they are all associated with a respiratory disease. Therefore, common genes affected by different viruses may also be useful for understanding the disease mechanism. For this purpose, the major genes commonly selected on different experiments are also obtained and provided as Supplementary file (Supplemental 3).

**Figure 6:**
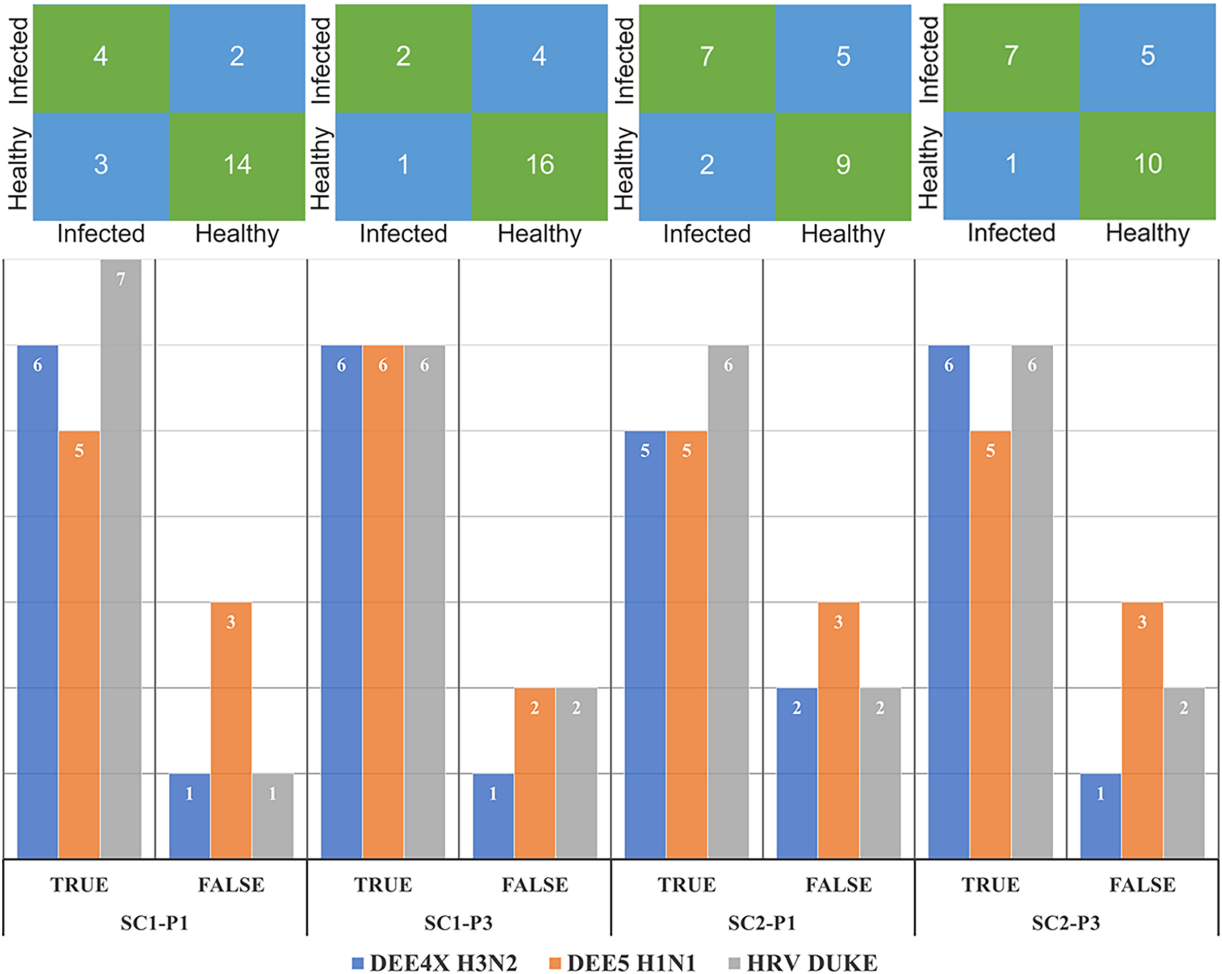
Confusion matrices and number of correctly and mis-classified subjects for each testing experiment predicted by the most accurate models in Tables 2 and 3. The y-axis of the matrices indicates the actual condition of the subjects (*i.e*., true labels).

The number of correctly and misclassified samples for each respiratory virus on the test set is shown in Fig. 6 with respect to sub-challenge and phase. The top section of this figure includes confusion matrices with rows representing true labels and columns denoting predicted labels. Our best performing models correctly predicted 18 out of 23 test samples for SC-1 phase 1 and SC-1 phase 3, which gives an overall accuracy of 78.26%. ReliefF with LR classifier and Fisher Score with KNN classifier and STPE pre-processing approach were used for these models, respectively. On the other hand, the best performing model for SC-2 phase 1 correctly predicted 16 out of 23 test samples with an accuracy of 69.57% and the best performing model for SC-2 phase 3 correctly predicted 17 out of 23 test samples, which corresponds to an accuracy of 73.91%.

In our last experiment, we compared our method with DeepFlu, which is based on deep neural networks and is published recently in the literature. DeepFlu was specifically developed to predict symptom severity, which corresponds to SC-2 and was applied to the datasets for DEE2 H3N2 and DEE3 H1N1. It should be noted that DeepFlu used a gene-annotated versions for both datasets, while we used probe-annotated versions. This resulted in different number of input features for prediction models. Additionally, DeepFlu utilized Leave-one Person Out (L1PO) cross-validation experiment on combined samples from T0 and T24 time points to evaluate performance separately on DEE2 H3N2 and DEE3 H1N1 datasets. In our model, we used the AF approach for preprocessing and the XGBoost algorithm in default hyper-parameter settings without feature selection for computing predictions. Since DeepFlu results were obtained with L1PO, our model is evaluated using the samples belonging to DEE2 H3N2 and DEE3 H1N1 experiments only using LOOCV to make a fair comparison. The results show that the our method achieved an AUPRC of up to 0.956, outperforming DeepFlu’s AUPRC of 0.76 in predicting the SC-2 label of DEE3 H1N1. On the other hand, our method obtained an AUPRC of 0.946 while DeepFlu could obtain 0.901 for predicting the SC-2 label of DEE2 H3N2. The results of both models are available in Table 5, which shows the best performing method for each performance metric and for each virus experiment. Based on these results, our method outperforms DeepFlu in all performance metrics for DEE3 H1N1 and DEE2 H3N2.

**Table 5:** Results of the comparative analysis between DeepFlu and our method for DEE3 H1N1 and DEE2 H3N2 experiments for different time points. Bold values denote scores of best-performing models according to different performance metrics.

Experiment	Accuracy	Sensitivity	Specificity	Precision	AUROC	AUPRC
**H1N1**						
*DeepFlu T0*	0.700	0.616	0.822	0.718	0.787	0.758
*AF-XGB T0*	**0.952**	**1.000**	**0.900**	**0.917**	0.900	0.458
*DeepFlu T24*	0.669	0.613	0.715	0.679	0.725	0.712
*AF-XGB T24*	0.857	0.909	0.800	0.833	**0.909**	**0.956**
**H3N2**						
*DeepFlu T0*	0.738	0.722	0.756	0.770	0.847	0.901
*AF-XGB T0*	**0.882**	**0.889**	**0.875**	**0.889**	**0.889**	**0.946**
*DeepFlu T24*	0.689	0.689	0.723	0.759	0.759	0.806
*AF-XGB T24*	0.765	0.778	0.750	0.778	0.778	0.888

## Discussion

In this study, we aimed to improve the accuracy of predicting infection and symptom development in individuals exposed to respiratory viruses by using different machine learning models and approaches. Our results were compared with the Respiratory DREAM Challenge, which is considered as one of the most important competitions in the field. Among the proposed approaches, STPE, which treats each time point separately, and AF, which combines gene expression at different time points, performed better than the Challenge leaderboard in all categories in terms of prediction. Although merging samples from the same virus to enlarge train dataset (*i.e*., the VM approach) improved the prediction performance for some of the tasks, this was not observed in all settings.

One of the interesting findings of the experiments is that although the accuracy of predicting whether a particular sample is infected increased in post-exposure for most of the models, a reverse behavior is observed for the remaining models. After inoculation of the virus into the body, some genes are expressed as part of the immune system against the infection. Therefore, profiling values of the expressed genes could be expected to be more discriminative for prediction. However, results show that the number of correctly predicted subjects did not increase for DEE4X H1N1 in SC-1 and DEE5 H3N2 in SC-2 even after exposure to respiratory virus. On the contrary, the prediction accuracy decreased after exposure in HRV DUKE experiments in the SC-1 category.

Another approach we have used in our experiments is FS, which achieved very good results despite using too few gene expression values. For example, the mRMR-AF based model achieved such a high AUPRC of 0.9325 even though only 16 gene expression feature values were used. This result shows that the majority of features in the dataset might be irrelevant or redundant, considering that the total number of features is 22,777.

Intersecting genes of SC-1 and SC-2 selected by the most successful FS methods (see Table 3) are found as ATP7A, HLA-DQA1, HLA-DRB4, XIST, LOC389906 in phase 1 and ATP7A and FCER1A in phase 3, respectively. All of these genes were mentioned as related to respiratory infection in the literature (Janssen et al., 2007; Boyton et al., 2008; Jong et al., 2016). Especially, “ATP7A” gene selected commonly for SC-1 and SC-2 is found to be related to virus replication process of influenza A (Rupp et al., 2017).

It can be observed that the number of genes jointly selected by the FS algorithms for the presence of viral shedding and the presence of symptoms is small. There could be several reasons for this result. The first reason could be related to selecting minimum number of features that give the best prediction performance during FS. The second reason is while calculating the number of jointly selected genes, we considered the FS methods which selected less than 100 features only. This is because we aimed to find out the top representative genes in terms of prediction performance for different sub-challenges and phases. When we also used other feature selection methods that selected more than 100 features, the intersection set was empty. The third reason for having a small number of intersecting genes might be related to selecting features for each phase and sub-challenge individually and then taking their intersections. When we examine Fig. 4, the total numbers of selected genes are not quite small in each cluster. For example, the number of jointly selected genes of only the SC1-Phase 3 is 104. However, when the intersections of multiple clusters are taken, the numbers that show common effective genes decrease considerably. The fourth reason could be related to having small number of samples in our dataset. Due to the small sample size, there could be noise and variance in the outputs of feature selection methods. This leads to ranking features differently for each feature selection method. The fifth reason could be due to sub-challenge differences. Although the same gene expression values are used for each sample, SC-1 aims to predict infection, while SC-2 aims to predict symptom severity. Therefore, sample-wise class labels were not always the same. For example, a subject could be labeled as infected but not labeled as showing symptoms, or *vice versa*. Due to differences in the labels, feature selection methods could select different genes for different sub-challenges.

As a further analysis to better understand the biological relationships of the selected genes, an ORA was performed for each category and phase separately. Pathways associated with “adaptive immune system” and “immune disease” were enriched in certain genes according to the results of ORA. In particular, the fact that selected genes in phase 1 were also associated with the immune system indicates that the immune system, the body’s defense mechanism against viruses, is also statistically critical for protection against respiratory infections. Moreover, according to the literature, genetic disorders of the adaptive and innate immune systems are one of the key factors responsible for repeated respiratory infections (Gibson et al., 2013; Lacoma et al., 2019).

## Conclusion

Respiratory infections are widespread, symptomatic, and contagious diseases that occur in all countries and regions of the world. Some people exposed to the virus are able to completely avoid infection, while others develop severe symptoms. To enhance the predictive performance for both infection and symptom severity, we sought to improve upon the results of the Respiratory DREAM Challenge, a significant competition in the field. Results show that our proposed approaches have improved the prediction of infection (up to 0.97 AUPRC) and symptom severity (up to 0.93 AUPRC) compared to the methods submitted to the challenge. Furthermore, analysis of the mutual genes selected by feature selection methods showed that the “immune system” has a strong association with symptom development. These findings also showed congruity with the biological studies in the literature.

In the next studies, the proposed approaches and methods will be performed on the other gene expression dataset collected with a different microarray chipset, *e.g*., from Illumina. The predominant genes will be investigated during symptomatic peak periods, considering gene expression up to 120 h. Furthermore, the Gene Set Enrichment Analysis (Subramanian et al., 2005) approach will be utilized to improve predictive performance and identify the most enriched pathways according to infection.

## Supplemental Information

10.7717/peerj.15552/supp-1 Supplemental Information 1 Hyper parameter space of machine learning algorithms.

10.7717/peerj.15552/supp-2 Supplemental Information 2 Z-TEST Significance comparison between our best results and the DREAM Challenge results.

10.7717/peerj.15552/supp-3 Supplemental Information 3 Selected common genes from different experiments.

## Additional Information and Declarations

### Data Availability

The following information was supplied regarding data availability: The data are available at GEO: GSE73072. The matrix files (SOFT, Minimal, *etc*.) were generated using only the gene information. In our experiment, we used probes rather than genes. Therefore, the data should be regenerated using the correct CDF (annotation file) and raw files (.CEL, files are available on GEO). Detailed information about the re-generation and the regenerated dataset are available in GitHub: https://github.com/yeisik/respiratory_infection_prediction.
