# Supplementary material for: Comparative analysis of machine learning approaches for predicting respiratory virus infection and symptom severity
Source: PeerJ. 2023 Jun 30;11:e15552. doi: 10.7717/peerj.15552 (PMC10317018; doi:10.7717/peerj.15552)
Supplement: Supplemental Information 1 [file peerj-11-15552-s001.pdf]

Table 1. Optimized hyperparameters and value ranges for regression algorithms

Bayesian Ridge	Alpha 1	(0.0000001 - 1)
	Alpha 2 (Inverse Scale Alpha)	(0.0000001 - 1)
	Lambda 1	(0.0000001 - 1)
	Lambda 2 (Inverse Scale Lambda)	(0.0000001 - 1)
DecisionTreeRegressor	Splitter	[Best, Random]
ElasticNet	Alpha	(0.0001 - 2)
	L1 Ratio	(0.05 - 1)
GradientBoostingRegressor	Learning Rate	(0.01 - 1)
	Number of estimators	(50 - 1000)
	Subsample	(0.001 - 1)
KNeighborsRegressor	Number of estimators	(2 - Number of Samples)
	Weights	[Uniform, Distance]
	Algorithm	[Ball Tree, KD Tree, Brute]
	Leaf Size	(10 - 100)
	Power	(1 - 3)
Lasso	Alpha	(0.0001 - 2)
	Selection	[cyclic, random]
LinearSVR	Epsilon	(0 - 1)
	Regularization (C)	(0.00001 - 1000)
RandomForestRegressor	Number of estimators	(50 - 1000)
Ridge	Alpha	(0.0000001 - 1)
	Solver	[svd, cholesky, lsqr, sparse cg, sag, saga]
XGBRegressor	Booster	[GBTree, GBLinear, Dart]
	Learning Rate (eta)	(0.001 - 1)
	Gamma	(0 - 100)
	Max Depth	(2 - 20)
	Subsample	(0.0001 - 1)
	Lambda	(0.01 - 5)
	Alpha	(0.01 - 5)

Table 2. Optimized hyperparameters and value ranges for classification algorithms

Logistic Regression	Regularization (C)	(0.0001 - 4000)
	Solver	[LibLinear, LBFGS, Newron-cg, Saga]
Support Vector Machines	Regularization (C)	(0.0001 - 4000)
	Kernel	[Linear, Poly, RBF]
	Gamma	(0.00001 - 10)
Random Forest	Number of Estimators	(2 - 1000)
	Split Criteria	[Gini, Entropy]
	Min Split Samples	(1 - 32)
k-Nearest Neighbors	Number of Neighbors	(2 - Number of Samples)
	Algorithm	[Auto, Ball Tree, KD Tree, Brute]
XGBoost	Booster	[GBTree, GBLinear]
	Number of Estimators	(2 - 1000)
	Max Depth	(2 - 512)
	Learning Rate (eta)	(0.0001 - 10)
	Gamma	(0 - 100)
	Alpha	(0 - 32)
	Lambda	(0 - 32)
