# Supplementary material for: Comparative analysis of machine learning approaches for predicting respiratory virus infection and symptom severity
Source: PeerJ. 2023 Jun 30;11:e15552. doi: 10.7717/peerj.15552 (PMC10317018; doi:10.7717/peerj.15552)
Supplement: Supplemental Information 2 [file peerj-11-15552-s002.pdf]

Tablo 1. Z-TEST Significance comparison between our best results and the DREAM Challenge results.

Z-TEST	SC1 T0 (ReliefF*)	SC1 T24 (Fisher Score*)	SC2 T0 (AF*)	SC2 T24 (AF*)	SC3 T0 (F Statis.)	SC3 T24 (F Statis.*)
**Z-Score**	0.1244	0.7121	0.6207	1.5254	0.4482	1.1934
**P value**	0.4522	0.2388	0.2676	0.0630	0.3263	0.1170
