# Supplementary figures and images for: Comparative analysis of machine learning approaches for predicting respiratory virus infection and symptom severity
Source: PeerJ. 2023 Jun 30;11:e15552. doi: 10.7717/peerj.15552 (PMC10317018; doi:10.7717/peerj.15552)

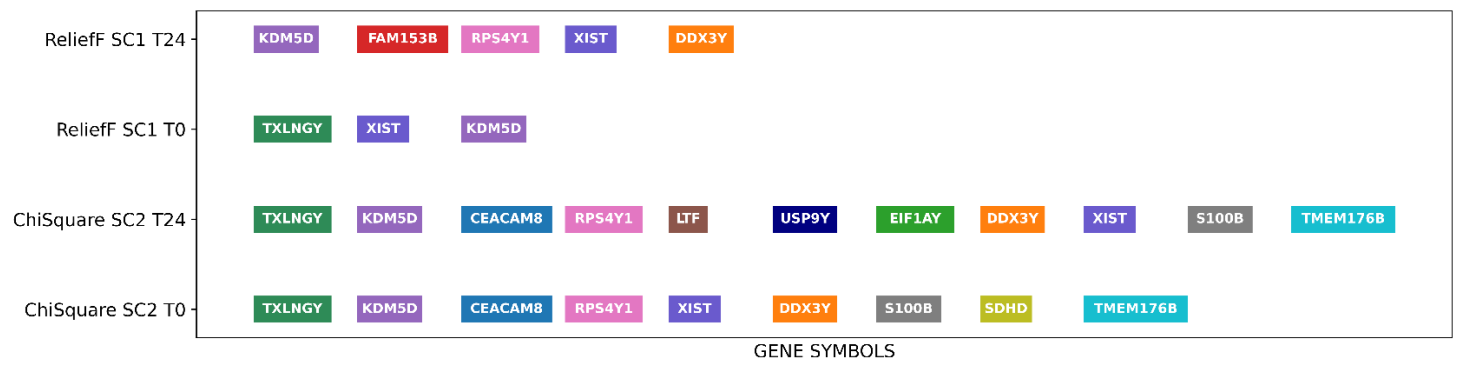

Figure 1 . Genes commonly selected for different experiments

Supplement: Supplemental Information 3 [file peerj-11-15552-s003.pdf]
